# Network Pharmacology and Transcriptome Analysis Reveal Potential Cardiometabolic Targets of *Polygonum cuspidatum*

**DOI:** 10.3390/biomedicines14030516

**Published:** 2026-02-26

**Authors:** Jihong Oh, Jieun Choo, Garam Yang, Hongmin Chu, Won G. An

**Affiliations:** 1 Mapo Hongik Korean Medicine Clinic, Seoul 04173, Republic of Korea; jihong421@gmail.com (J.O.); hongminchu2@gmail.com (H.C.); 2 Usher Bio Co., Seoul 04172, Republic of Korea; jiun7788@naver.com (J.C.); gr0011@hanmail.net (G.Y.); 3 Incheon Gangchoo Korean Medicine Clinic, Incheon 21565, Republic of Korea; 4 Department of Pharmacology, College of Korean Medicine, Pusan National University, 49 Pusandaehak-ro, Mulgeumeup, Yangsan 50612, Republic of Korea

**Keywords:** *Polygonum cuspidatum*, network pharmacology, transcriptomics, insulin resistance, atherosclerosis, inflammatory–oxidative pathways

## Abstract

**Objectives**: *Polygonum cuspidatum* Sieb. et Zucc (PC) has traditionally been used for inflammatory and circulatory disorders; however, the systems-level mechanisms of its effect on cardiometabolic disease processes, including insulin resistance and vascular injury, remain incompletely understood. This study aimed to identify biological pathways potentially modulated by PC through the integration of network pharmacology with patient-derived transcriptomic data. **Methods**: Four representative compounds—resveratrol, polydatin, emodin, and physcion—were selected based on previously reported chemical fingerprints that characterize PC. Predicted targets were obtained from public compound–target databases and used to construct a compound–target network. Functional enrichment was performed using Kyoto Encyclopedia of Genes and Genomes (KEGG) pathway analysis and Genetic Association Database (GAD) disease associations. To evaluate clinical relevance, predicted targets were compared with differentially expressed genes (DEGs) from insulin-resistant adipose tissue (GSE20950) and atherosclerotic lesions (GSE43292). **Results**: A total of 329 predicted target genes were identified, with resveratrol emerging as the dominant topological hub (214 targets). Network and enrichment analyses highlighted *MAPK14*, *MAPT*, *VEGFA*, *IL1B*, *NLRP3*, and *HMOX1* as key targets involved in inflammatory, oxidative, and vascular injury pathways that overlapped with transcriptomic signatures. KEGG analysis demonstrated significant enrichment in AGE–RAGE signaling, TNF-mediated inflammation, and lipid–atherosclerosis pathways, while GAD mapping indicated associations with type 2 diabetes and atherosclerosis. Integration of transcriptomic datasets further supported a convergence on coordinated inflammatory and oxidative processes driving vascular remodeling. **Conclusions**: These findings suggest that the major constituents of PC may modulate interconnected cardiometabolic processes linking insulin resistance and vascular injury implicated in atherosclerotic cardiovascular disease. By integrating network pharmacology with patient-derived transcriptomic evidence, this study provides a systems-level framework for interpreting the potential biological roles of PC in insulin resistance and vascular injury.

## 1. Introduction

Cardiometabolic diseases—a cluster of interrelated conditions including obesity, type 2 diabetes, dyslipidemia, and hypertension—represent a significant global health burden [1]. These disorders are characterized by shared pathogenic mechanisms, such as oxidative stress, chronic inflammation, and endothelial dysfunction [2,3,4,5]. Because these disorders arise from multifactorial and interconnected processes, single-target pharmacotherapies often provide limited benefit [6,7]. This has led to increasing interest in natural products capable of modulating complex metabolic and vascular networks through multi-component mechanisms [8].

*Polygonum cuspidatum* Sieb. et Zucc. (PC), a widely used herbal medicine, has traditionally been prescribed for inflammatory and circulatory conditions. Classical records describe its actions as promoting blood circulation, alleviating inflammation, and supporting detoxification, functions that broadly correspond to improving microcirculation and reducing inflammatory responses in modern pharmacological terms. Contemporary studies further report antioxidant, anti-inflammatory, antimicrobial, and metabolic effects, suggesting potential relevance to cardiometabolic disorders [9,10,11].

In this study, PC refers specifically to its medicinal root and rhizome (*Polygoni Cuspidati Rhizoma et Radix*), which constitute the primary plant tissues used in clinical practice and pharmacological studies. PC is characterized by abundant stilbene and anthraquinone derivatives, including resveratrol, polydatin, emodin, and physcion, which contribute its characteristic chemical fingerprints [12,13]. These compounds have been shown to influence glucose and lipid metabolism and to modulate oxidative and inflammatory signaling pathways such as AMPK, SIRT1, NF-κB, JAK/STAT, and MAPK [14,15,16,17,18]. However, despite extensive investigation of individual constituents, the integrated, system-level mechanisms through which these combined bioactive compounds may exert biological effects relevant to metabolic and vascular disease processes remain insufficiently understood.

**Figure 1:**
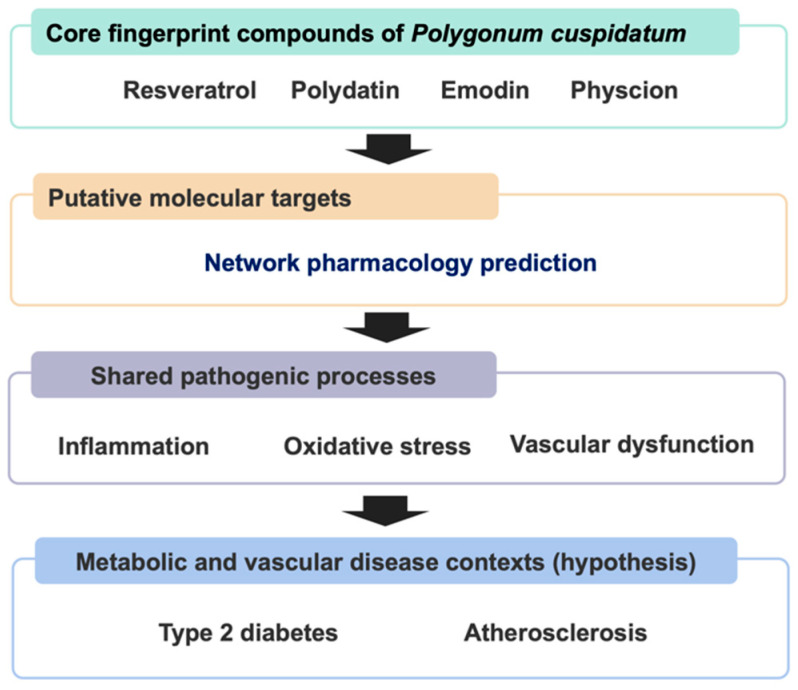
Conceptual framework of *Polygonum cuspidatum* linking predicted molecular targets to shared pathogenic processes across metabolic and vascular disease contexts.

This study aimed to characterize the molecular targets of PC by focusing on its four principal fingerprint constituents—resveratrol, polydatin, emodin, and physcion. Guided by the conceptual framework in Figure 1, we examined whether the predicted targets of these compounds converge on an integrated inflammatory–oxidative–vascular axis relevant to metabolic and vascular disease processes, such as type 2 diabetes and atherosclerosis. By integrating network pharmacology with patient-derived transcriptomic signatures, we sought to delineate a candidate set of genes to guide future validation in experimental models of metabolic and vascular disease. Ultimately, this study offers a data-driven framework to support hypothesis generation and rational experimental design for investigating biological relevance of PC across interconnected metabolic and vascular disease contexts.

## 2. Materials and Methods

### 2.1. Core Compounds of PC

To identify the chemical constituents of PC, core bioactive compounds were selected based on prior phytochemical and pharmacological studies. Four representative compounds—resveratrol, polydatin, emodin, and physcion—were defined as the “Core-4” compounds due to their well-established biological activities and relevance to the therapeutic effects of PC [19].

Pharmacokinetic parameters, including oral bioavailability (OB), drug-likeness (DL), Caco-2 permeability, and molecular weight, were retrieved from the Traditional Chinese Medicine Systems Pharmacology (TCMSP version 2.3, accessed on 21 January 2026) database by querying “虎杖 (Huzhang, *Polygoni Cuspidati Rhizoma et Radix*)”, in order to provide reference ADME information [20]. Although OB and DL thresholds are commonly applied as screening criteria in network pharmacology studies, they were not used as exclusion filters in the present analysis. This decision was based on the fact that the Core-4 compounds were pre-selected according to consistent experimental and pharmacological evidence and that predicted ADME parameters may underestimate the relevance of well-characterized phytochemicals. Accordingly, TCMSP-derived OB and DL values were used for descriptive purposes only. To further characterize the physicochemical drug-likeness of the core compounds from a complementary perspective, DL was additionally evaluated according to Lipinski’s rule-of-five using the HERB 2.0 database.

### 2.2. Target Proteins and Genes

The molecular targets of the Core-4 compounds were identified using the HERB 2.0 database (http://herb.ac.cn/v2, accessed on 21 January 2026) [21]. For each compound, searches were conducted based on an exact match of the InChIKey obtained from the TCMSP database, with ingredient names, aliases, and CAS numbers or TCMSP identifiers used for additional verification.

Only target genes supported by reference-mining evidence in HERB 2.0 were collected. Corresponding gene symbols were standardized, and duplicate entries were removed prior to downstream analyses. The resulting compound–target associations were compiled into a structured dataset for subsequent enrichment and network analyses.

### 2.3. Pathway and Disease Enrichment Analysis of Target Genes

Enrichment analysis of the predicted target genes of PC was performed to identify biological pathways and disease categories potentially modulated by the Core-4 compounds. KEGG pathway enrichment was conducted using g:Profiler (https://biit.cs.ut.ee/gprofiler/gost, accessed on 23 January 2026) [22], applying the Benjamini–Hochberg false discovery rate (BH FDR) as a multiple testing correction method. Pathways with an adjusted *p*-value (BH FDR) < 0.01 were considered statistically significant. Disease association analysis was performed using the GAD module implemented in DAVID 2021 (https://davidbioinformatics.nih.gov/, accessed on 23 January 2026) [23,24]. Statistical significance was defined as an adjusted *p*-value (BH FDR) < 0.01 [25].

### 2.4. Network Construction and Visualization

Compound–target gene interaction networks were constructed and visualized using Cytoscape (version 3.10.4) [26]. Nodes represented compounds and target genes, while edges indicated compound–target interactions. Shared targets among the Core-4 compounds were used to identify hub genes. Network topological parameters, including degree and betweenness centrality, were calculated to identify hub nodes.

### 2.5. DEG Analysis Based on Patient Transcriptomic Data

To examine the clinical relevance of the predicted target genes, differentially expressed gene (DEG) analyses were performed using human transcriptomic datasets associated with cardiometabolic disorders. Gene expression profiles were retrieved from the Gene Expression Omnibus (GEO) database (https://www.ncbi.nlm.nih.gov/geo/, accessed on 23 January 2026) by applying the following search parameters: (“diabetes mellitus” OR “atherosclerosis”) AND “Homo sapiens” [Organism] AND “Expression profiling by array” [Filter] [27,28]. Datasets were screened according to the following criteria:

- Presence of a clearly defined case–control comparison;
- Sample size ≥ 15 per group;
- Availability of complete platform and annotation files;
- Exclusion of studies using peripheral blood mononuclear cells (PBMCs) to focus on disease-relevant tissues.

Two datasets met these selection criteria: GSE43292, which profiles carotid artery atheromatous plaques (*n* = 32) and matched intact arterial tissue (*n* = 32) from hypertensive patients [29], and GSE20950, which profiles subcutaneous and visceral adipose tissue from BMI-matched obese individuals who were insulin-resistant (*n* = 19) versus insulin-sensitive (*n* = 20), thereby enabling evaluation of insulin resistance independently of obesity [30,31].

DEG analysis was conducted using the GEO2R tool, which applies a linear-model-based framework implemented in the limma package (version 3.54.0). Log_2_ transformation was handled using GEO2R’s automatic detection option. Voom precision weights were applied, and multiple testing correction was performed using the BH FDR method. Significant DEGs were defined using the following thresholds: adjusted *p*-value < 0.01 and |log_2_ fold change (FC)| > 0.58.

Probe identifiers were mapped to official gene symbols according to each dataset’s annotation file. Probes lacking valid gene symbols or containing ambiguous entries were removed. Overlap analysis was then performed between DEGs from each dataset (GSE20950, GSE43292) and the target genes of the Core-4 compounds of PC. Shared genes were used for subsequent KEGG pathway enrichment, conducted with g:Profiler, to identify cardiometabolic disease-related pathways involving PC-associated targets.

Volcano plots, Venn diagrams, and dot plots were generated in Python (version 3.12.12) using matplotlib (ver 3.10.0), seaborn (ver 0.13.2), pandas (ver 2.0.2), numpy (ver 2.0.2), scipy (1.16.3), and matplotlib-venn3 (ver 1.1.2). Volcano plots were generated at the probe level, while downstream gene-level analyses (Venn diagrams and pathway enrichment) were performed after collapsing multiple probes per gene by selecting the probe with the largest |log_2_ FC|.

### 2.6. Pathway Enrichment Validation

To assess pathway-level alterations within the full transcriptomic background, global KEGG enrichment analysis was performed using g:Profiler with dataset-specific custom backgrounds consisting of all quality-controlled genes. Statistical significance was defined as BH FDR < 0.01.

Targeted over-representation analysis was conducted for the AGE–RAGE signaling pathway (KEGG:04933) and the lipid and atherosclerosis pathway (KEGG:05417), using one-tailed Fisher’s exact test (SciPy 1.16.3), with significance defined as *p* < 0.05.

Gene-level expression changes for pathway-associated DEGs were visualized as log_2_ fold-change heatmaps using genes meeting predefined differential expression criteria (adjusted *p* < 0.01 and |log_2_FC| > 0.58). Pathway gene annotations were retrieved from the KEGG database (https://www.kegg.jp/kegg/, Release 117.0, 1 January 2026).

## 3. Results

### 3.1. ADME Characteristics of the Core-4 Compounds

The absorption, distribution, metabolism, and excretion (ADME)-related physicochemical properties of the four representative marker compounds of PC—resveratrol, polydatin, emodin, and physcion—were analyzed for their pharmacokinetic properties using the TCMSP database (Table 1). All compounds had molecular weights below 500 Da and Caco-2 permeability values greater than −0.2, indicating acceptable intestinal absorption potential. Their oral bioavailability (OB) values were relatively low and did not meet the conventional threshold commonly applied in network pharmacology studies (OB ≥ 35%). In contrast, TCMSP-derived drug-likeness (DL) values exceeded the commonly used cutoff (DL ≥ 0.18) for three compounds—polydatin, emodin, and physcion—while resveratrol showed a lower DL value. Despite the relatively low OB values, the four compounds were retained for subsequent analyses owing to their well-documented pharmacological relevance and characteristic presence in PC. To further characterize their physicochemical drug-likeness from a complementary perspective, Lipinski’s rule-of-five compliance was evaluated using the HERB 2.0 database (Supplementary Table S1). Three compounds fully satisfied all Ro5 criteria, while polydatin showed a single Ro5 violation, indicating overall acceptable drug-like properties among the Core-4 compounds.

To contextualize the selection of these four fingerprint compounds from a pharmacognostic perspective, previously reported quantitative analyses of Polygonum cuspidatum tissues were compiled (Table 2). Independent studies have consistently demonstrated that the medicinal root and rhizome contain markedly higher concentrations of stilbene and anthraquinone derivatives compared to aerial parts [11]. In particular, polydatin and emodin are present in the root at milligram-per-gram levels, whereas their contents in stem and leaf tissues are minimal or frequently below detection limits [32,33]. This tissue-specific enrichment supports the designation of the root and rhizome as the principal medicinal sources and provides a pharmacognostic rationale for focusing on these fingerprint compounds in subsequent system-level analyses.

### 3.2. Target Gene Prediction of the Core-4 Compounds

**Figure 2:**
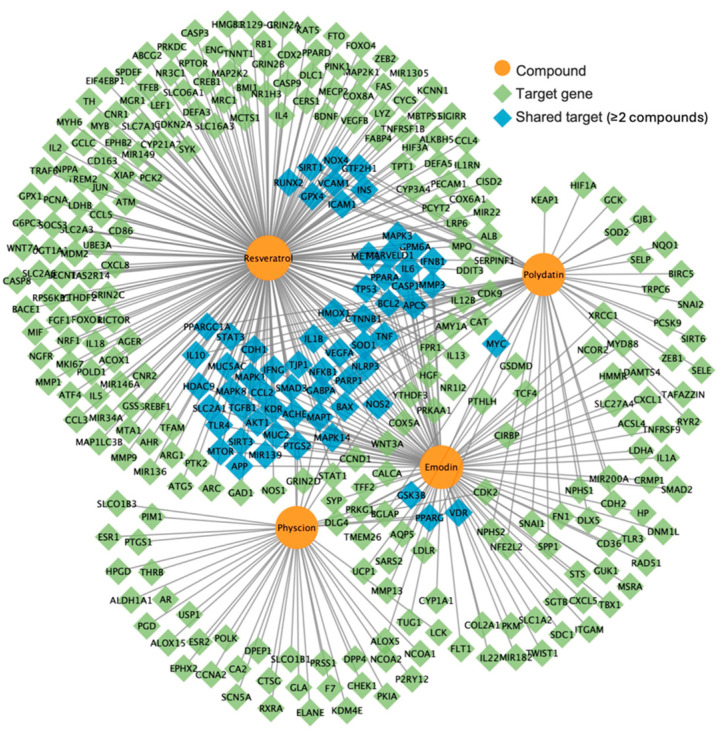
Compound-target gene network of the Core-4 compounds of *Polygonum cuspidatum*. Orange circles represent the core compounds (resveratrol, polydatin, emodin, and physcion), and diamond-shaped nodes represent predicted target genes. Blue diamonds indicate target genes shared by two or more compounds, whereas green diamonds indicate targets associated with a single compound. Edges represent compound–target interactions.

A total of 329 non-redundant target genes were identified across the Core-4 compounds (Figure 2). Among them, resveratrol was associated with the largest number of targets (214), followed by emodin (96), polydatin (54), and physcion (43).

Target overlap analysis showed that NOS2 was the only gene shared by all four compounds. In addition, fourteen genes were shared by three compounds, whereas 47 genes were common to two compounds (Table 3). Pairwise comparison revealed that emodin and resveratrol shared the largest number of targets (43 genes), followed by resveratrol and polydatin (25 genes), indicating the broad target coverage of resveratrol within the network. Network topology analysis identified *NOS2*, *BAX*, *MAPK14*, *MAPT*, and *PTGS2* as key hub genes based on degree and betweenness centrality measures (Table 3).

### 3.3. KEGG Pathway Enrichment Analysis

**Figure 3:**
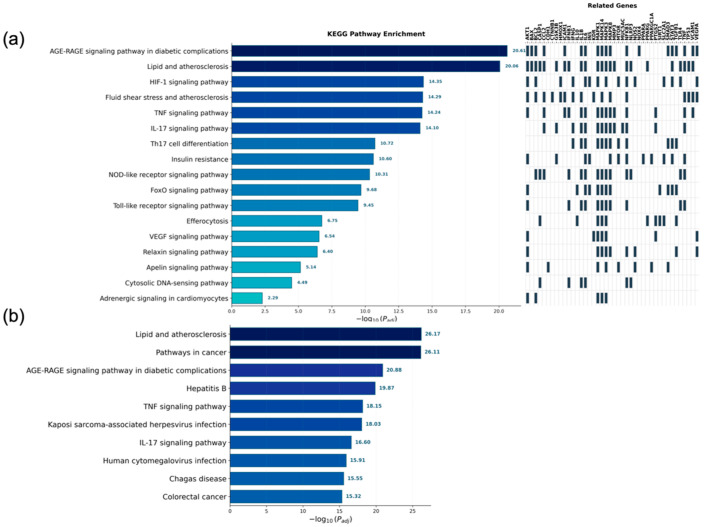
KEGG pathway enrichment for hub genes and all target genes of *Polygonum cuspidatum*. (**a**) Significant KEGG pathways (adjusted *p* < 0.01) enriched from the hub genes are listed, with representative metabolic, vascular, and inflammation-related pathways such as AGE–RAGE signaling in diabetic complications, lipid and atherosclerosis, HIF-1, TNF signaling pathway, and insulin resistance; (**b**) For the full target gene set, the top 10 enriched pathways are shown among those satisfying adjusted *p* < 0.01, ranked by −log_10_ (adjusted *p*).

Genes associated with two or more of the Core-4 compounds were used for KEGG pathway enrichment analysis. Significant pathways were identified using g:Profiler with thresholds of adjusted *p* < 0.01. Among the enriched pathways, several were categorized as metabolic, vascular, and inflammation-related pathways, including AGE–RAGE signaling in diabetic complications, lipid and atherosclerosis, TNF signaling, IL-17 signaling, and insulin resistance (Figure 3a). These pathways encompassed multiple high-centrality genes identified from the compound–target network.

To compare these results with the broader target landscape, KEGG enrichment analysis was also performed for the full set of 329 target genes. The top 10 pathways, selected among those meeting the same statistical thresholds and ranked by −log_10_ (adjusted *p*), are presented in Figure 3b. Enriched pathways in the full target gene set similarly included cardiometabolic and inflammation-related categories.

### 3.4. Disease Association Analysis

**Figure 4:**
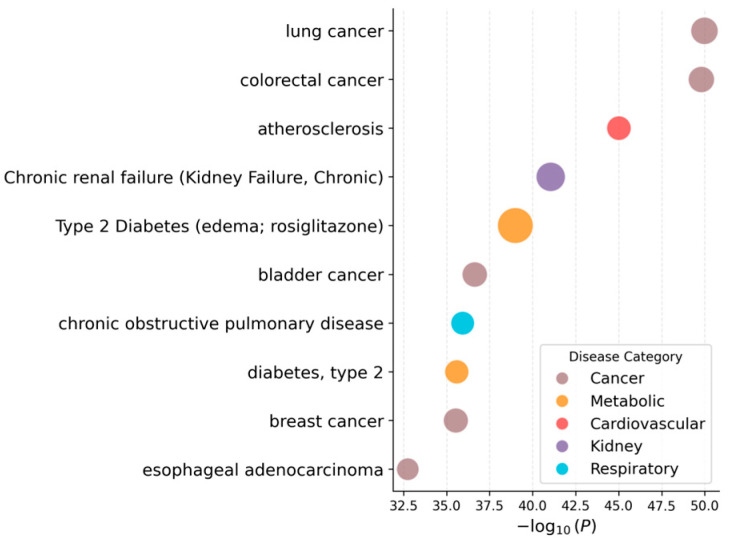
GAD disease enrichment analysis for the predicted target genes of *Polygonum cuspidatum*. Results are presented as a dot plot for the top 10 enriched disease terms. Dot size indicates gene count, and dot color represents disease category. The *x*-axis shows −log_10_ adjusted *p* values.

Disease enrichment analysis was performed using the target genes through the Genetic Association Database (GAD) module in DAVID 6.8. The top 10 enriched disease categories, ranked by −log_10_ (*p*), are summarized in Figure 4. To further assess the robustness of disease association results across different databases, an additional disease enrichment analysis was conducted using DisGeNET, and the top 20 enriched diseases are presented in Supplementary Figure S1. The enriched categories included multiple cancer-related terms (e.g., lung, colorectal, bladder, prostate, and breast cancer) as well as metabolic disorders such as atherosclerosis, and type 2 diabetes mellitus.

### 3.5. DEG in Adipose and Vascular Tissues

#### 3.5.1. DEG Identification in Adipose and Vascular Tissues

**Figure 5:**
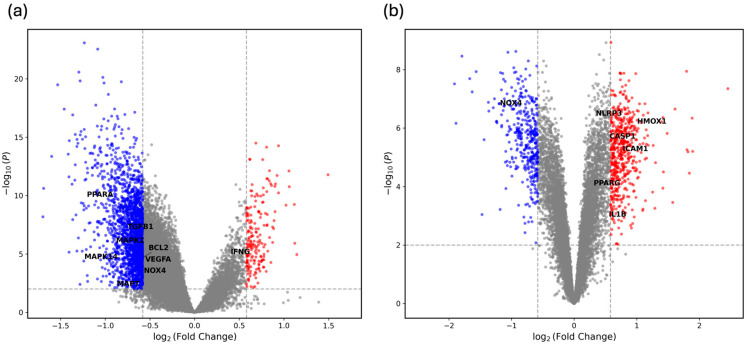
Volcano plots of differential gene expression from two transcriptomic datasets. (**a**) GSE20950 (insulin-resistant vs. insulin-sensitive adipose tissue); (**b**) GSE43292 (atheroma plaque vs. intact arterial tissue). Volcano plots were generated for both datasets using |log_2_FC| > 0.58 and adjusted *p* < 0.01 as significance thresholds. Upregulated genes (red), downregulated genes (blue), and non-significant genes (grey) are indicated. Labeled genes denote *Polygonum cuspidatum* target genes that overlapped with significant DEGs and mapped to KEGG pathways related to metabolic and vascular disease processes.

Differential gene expression analysis was performed for the GSE20950 and GSE43292 datasets using significance thresholds of |log_2_ FC| > 0.58 and adjusted *p* < 0.01. In GSE20950 (insulin-resistant vs. insulin-sensitive adipose tissue), 178 upregulated and 1819 downregulated probes were identified (Figure 5a). In GSE43292 (atheroma plaque vs. intact arterial tissue), 508 probes were upregulated and 369 were downregulated under the same criteria (Figure 5b).

#### 3.5.2. Intersection with *Polygonum Cuspidatum* Targets

**Figure 6:**
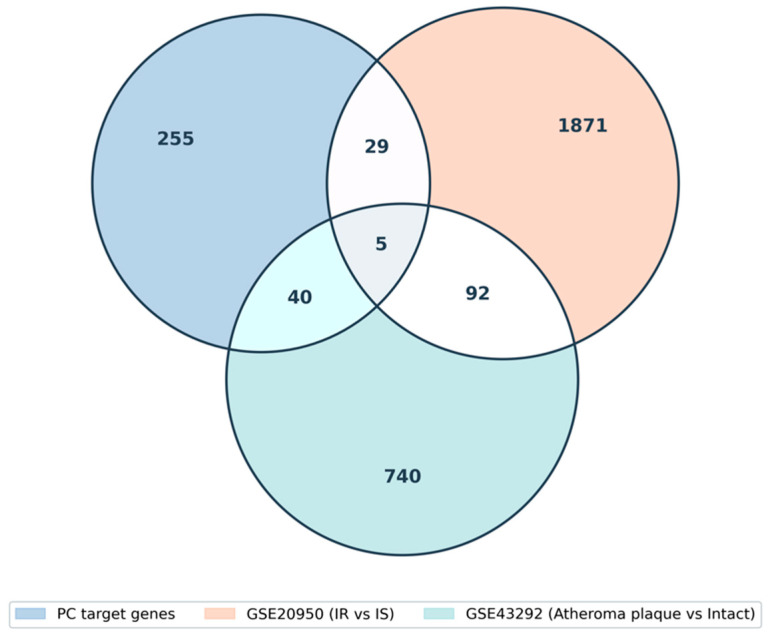
Overlap between *Polygonum cuspidatum* target genes and DEGs from GSE20950 and GSE43292. The Venn diagram displays the number of shared and unique genes across three datasets: predicted target genes of *Polygonum cuspidatum*, DEGs from GSE20950 (insulin-resistant vs. insulin-sensitive), and DEGs from GSE43292 (atheroma plaque vs. intact tissue). Overlapping regions indicate common differentially expressed genes among the datasets.

Intersection analysis revealed five genes shared among the Core-4 target set and both DEG datasets (*NOX4*, *PGD*, *PTGS1*, *FLT1*, and *ZEB1*) (Figure 6). In addition, 34 genes were shared with GSE20950 and 45 with GSE43292 (Supplementary Tables S2 and S3). Among these shared genes, *NOX4*, *MAPK14*, *MAPT*, *VEGFA*, *NLRP3*, *IL1B*, and *HMOX1* were targeted by multiple Core-4 compounds (Table 3).

#### 3.5.3. KEGG Pathway Enrichment of Shared Genes

**Figure 7:**
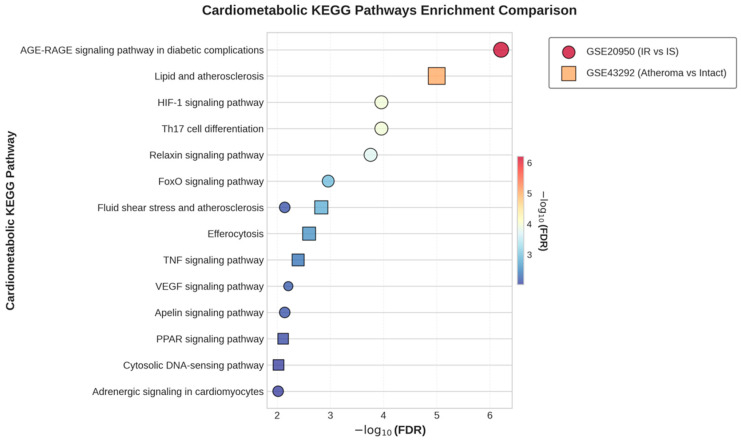
Selected KEGG pathway enrichment of genes shared between the Core-4 targets and DEGs from GSE20950 and GSE43292. Dot plots display significantly enriched KEGG pathways related to metabolic, inflammatory, and vascular processes derived from overlapping gene sets between predicted Core-4 targets and dataset-specific DEGs. Only pathways meeting the significance threshold (BH FDR < 0.01) are shown. Pathways are ranked by −log_10_(FDR), with dot size and color indicating the magnitude of enrichment.

KEGG pathway enrichment analysis was conducted for the overlapping gene sets between the predicted targets of the Core-4 compounds and the DEGs identified in each dataset. Metabolic, vascular, and inflammation-related pathways meeting the significance threshold (BH-FDR < 0.01) are summarized in Figure 7.

In GSE20950, the AGE–RAGE signaling pathway in diabetic complications showed the strongest enrichment signal. This was followed by pathways associated with hypoxia and immune regulation, including HIF-1 and Th17 cell differentiation, as well as signaling pathways related to vascular and metabolic regulation, such as relaxin signaling, FoxO signaling, and fluid shear stress and atherosclerosis.

In GSE43292, the lipid and atherosclerosis pathway exhibited the highest enrichment score, accompanied by additional significantly enriched pathways involved in vascular homeostasis and inflammatory responses, including fluid shear stress and atherosclerosis, efferocytosis, and TNF signaling.

#### 3.5.4. Quantitative Validation of Selected KEGG Pathways

Because the AGE–RAGE signaling and lipid/atherosclerosis pathways represented the top categories in the overlap-based KEGG enrichment (Figure 7), we performed additional analyses to quantify their enrichment strength within the full DEG background of each dataset.

Global KEGG enrichment of the complete DEG sets showed that the lipid and atherosclerosis pathway (hsa05417) ranked 31st in GSE43292 (*p* = 2.28 × 10^−4^), while the AGE–RAGE signaling pathway (hsa04933) ranked 64th (*p* = 9.65 × 10^−3^). In GSE20950, no KEGG pathways passed the BH-FDR < 0.01 threshold. However, AGE–RAGE ranked 34th with nominal enrichment (*p* = 1.46 × 10^−1^), indicating detectable but moderate pathway-level involvement (Supplementary Table S4).

**Figure 8:**
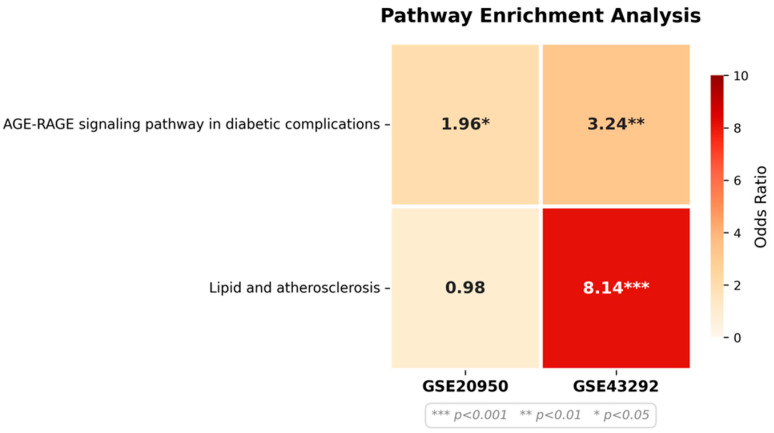
Targeted enrichment of AGE-RAGE signaling pathway in diabetic complications and lipid/atherosclerosis pathway. Heatmap showing odds ratios from Fisher’s exact test for AGE–RAGE signaling (hsa04933) and lipid and atherosclerosis (hsa05417) pathways in GSE20950 and GSE43292. Asterisks indicate statistical significance (* *p* < 0.05, ** *p* < 0.01, *** *p* < 0.001). Differentially expressed genes were defined as adjusted *p* < 0.01 and |log_2_FC| > 0.58.

We next assessed pathway-specific over-representation using Fisher’s exact test (Figure 8). In GSE43292, 9 of 67 AGE–RAGE genes were differentially expressed (13.4%; OR = 3.24, *p* = 3.49 × 10^−3^), and 17 of 61 lipid pathway genes were altered (27.9%; OR = 8.14, *p* = 1.29 × 10^−9^). In GSE20950, 12 of 70 AGE–RAGE genes were altered (17.1%; OR = 1.96, *p* = 3.30 × 10^−2^), whereas lipid pathway enrichment was not significant (6/64 genes; OR = 0.98, *p* = 5.84 × 10^−1^).

**Figure 9:**
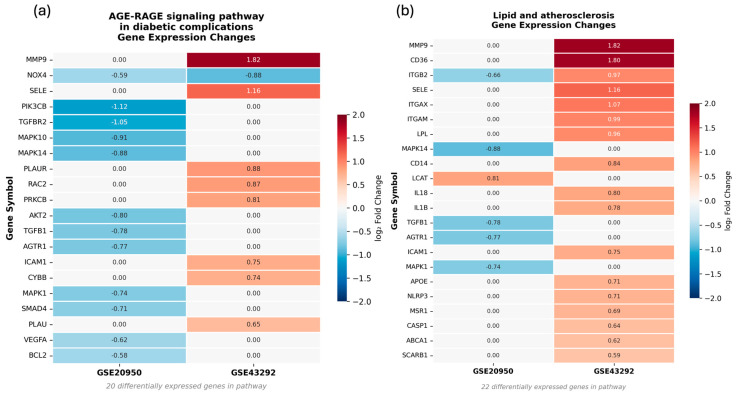
Gene-level expression changes in enriched cardiometabolic pathways. Heatmaps showing log_2_ fold changes for differentially expressed genes within (**a**) AGE-RAGE signaling pathway in diabetic complications and (**b**) lipid and atherosclerosis pathways across GSE43292 and GSE20950. Only genes meeting significance criteria (adjusted *p* < 0.01, |log_2_FC| > 0.58) are displayed.

Directionality analysis showed coordinated expression patterns at the pathway level (Figure 9). In GSE43292, lipid pathway genes were uniformly upregulated (17/17), and AGE–RAGE genes were predominantly upregulated (8/9). In contrast, AGE–RAGE genes in GSE20950 were consistently downregulated (12/12).

#### 3.5.5. Key Cardiometabolic Genes Targeted by Core-4 Compounds

Among the DEG–target intersections, eight genes were linked to two or more of the core-4 compounds and were also mapped to KEGG pathways associated with metabolic, inflammatory, and vascular processes (Table 4). In GSE20950, the overlapping genes *NOX4*, *MAPK14*, *VEGFA*, and *MAPT* were associated with multiple metabolic and vascular signaling pathways, including AGE–RAGE signaling, lipid and atherosclerosis, inflammatory signaling (TNF and IL-17), and metabolic regulation pathways such as FoxO and MAPK signaling. In GSE43292, *IL1B*, *ICAM1*, *NLRP3*, and *NOX4* were enriched predominantly in AGE–RAGE signaling, lipid and atherosclerosis, inflammatory signaling (TNF and IL-17), and fluid shear stress-related pathways.

## 4. Discussion

PC is characterized by multiple bioactive stilbenes and anthraquinones that collectively function via multi-component, multi-target mechanisms. Four representative fingerprint constituents—resveratrol, polydatin, emodin, and physcion—were selected because they constitute major pharmacologically active markers of PC. Although antioxidant and anti-inflammatory activities of these compounds are well documented, their integrated cardio-metabolic effects remain incompletely understood. Across network-derived targets and patient transcriptomic signatures, a consistent axis characterized by *MAPK14*, *MAPT*, *VEGFA*, *IL1B*, *NLRP3*, and *HMOX1* emerged as a convergent regulatory framework through which PC may modulate metabolic and vascular disease processes [35,36].

Metabolic and vascular disorders arise along a biological continuum in which clinical predictors—such as insulin resistance, dyslipidemia, and hypertension—initiate downstream pathogenic processes including chronic inflammation, oxidative stress, mitochondrial dysfunction, and endothelial remodeling. Within this framework, the inflammatory–oxidative–vascular axis identified in our analysis provides a conceptual bridge linking insulin resistance-driven metabolic alterations to inflammatory and vascular mechanisms contributing to atherosclerosis and related vascular disease outcomes. These findings suggest that predicted PC targets are positioned within interconnected pathogenic pathways rather than isolated molecular events.

Topological analysis of the compound–target network indicated that the Core-4 compounds interact with targets broadly distributed across metabolic and inflammatory signaling systems. Among these constituents, resveratrol accounted for the largest proportion of predicted targets (214/329), occupying a dominant topological position within the network. Importantly, its relevance extended beyond target quantity; resveratrol-associated targets show significant overlap with transcriptomic signatures derived from insulin-resistant adipose tissue (GSE20950) and atherosclerotic lesions (GSE43292). This convergence indicates that resveratrol may function as a principal regulatory node linking predicted molecular interactions with transcriptional alterations observed in patient-derived disease contexts.

Network analysis further indicated that resveratrol and emodin shared the largest number of predicted targets (*n* = 43), including key inflammatory regulators such as *MAPK14* and *IL1B*. The convergence of multiple compounds on common signaling nodes suggests coordinated modulation of inflammatory pathways at the systems level. Although this observation does not establish pharmacological synergy, it supports the hypothesis that multi-component interactions within PC may contribute to pathway-level regulation and warrants future experimental validation.

Consistent with previously reported biological activities of resveratrol, several predicted targets identified in this study are functionally linked to signaling systems involved in metabolic and vascular regulation, including pathways associated with AMPK-mediated energy sensing, NF-κB-dependent inflammatory signaling, and Nrf2-driven oxidative stress responses [37,38]. While these pathways did not emerge as the top-tier enrichment categories in our primary analysis, their known interactions with key identified targets—such as *MAPK14*, *IL1B*, and *HMOX1*—support the biological plausibility of the predicted regulatory framework.

Several identified targets provide mechanistic context for the observed pathway convergence. Notably, *MAPK14* (p38 MAPK) has been shown to act upstream of inflammasome activation by modulating NLRP3 signaling, thereby linking cellular stress sensing to inflammatory effector responses [35,39]. This interaction provides a plausible mechanistic bridge between stress-responsive kinase signaling and inflammasome-driven inflammation relevant to both insulin resistance and vascular pathology.

*HMOX1* represents a well-characterized oxidative stress-responsive effector with established roles in cytoprotection, mitochondrial homeostasis, and endothelial adaptation [40,41]. Induction of *HMOX1* via the Nrf2 signaling pathway has been demonstrated for resveratrol and polydatin in vascular and metabolic cell models, supporting consistency between experimentally observed antioxidant effects and the mechanisms predicted by our network framework [37,42]. Through regulation of redox balance and mitochondrial stress responses, *HMOX1* may contribute to stabilization of cellular environments exposed to chronic inflammatory and metabolic stress [43].

Regarding vascular adaptation, *MAPT* (tau), although predominantly studied in neurodegenerative disease, has been implicated in cytoskeletal regulation, cellular stress adaptation, and autophagy-related processes [44,45]. These functions suggest a potential role in maintaining cellular structural integrity under inflammatory and oxidative stress conditions relevant to vascular remodeling.

Consistent with these mechanistic observations, previous experimental studies have independently reported lipid-lowering, cardioprotective, antioxidant, and anti-inflammatory effects of PC extracts and their major constituents in both in vitro and in vivo models [9]. However, these studies have largely examined individual biological outcomes in isolation, without defining how multi-component phytochemicals collectively converge on shared pathogenic mechanisms. The present network pharmacology–transcriptomic integration provides a systems-level framework that connects these previously reported effects through coordinated inflammatory, oxidative, and vascular signaling pathways.

However, pharmacokinetic considerations remain important when interpreting the biological relevance of the Core-4 compounds. Based on TCMSP-derived ADME parameters, three compounds—polydatin, emodin, and physcion—exhibited acceptable DL values, whereas resveratrol showed lower DL despite its well-established bioactivity. Evaluation against Lipinski’s rule of five indicated that resveratrol, emodin, and physcion largely conform to conventional small-molecule criteria, while polydatin deviates due to its glycosylated structure and higher molecular weight. Notably, all four compounds displayed moderate oral bioavailability (OB, ~19–24%), suggesting that systemic exposure following oral administration may be constrained by metabolic factors typical of polyphenolic natural products. For example, resveratrol exhibits high intestinal absorption but low circulating concentrations owing to rapid first-pass metabolism [46], and emodin similarly shows limited bioavailability related to solubility and metabolic instability [47,48].

Recent advances in formulation strategies, including fermentation-based preprocessing and optimized delivery systems, have been reported to partially overcome the limited solubility and bioavailability of stilbene and anthraquinone derivatives [49,50]. In particular, fermentation may enhance bioaccessibility by converting glycosylated compounds such as polydatin into aglycones like resveratrol, thereby facilitating earlier intestinal absorption and reducing reliance on variable gut microbiota-mediated metabolism [51,52]. Although such approaches do not eliminate rapid metabolic clearance, they may increase systemic exposure to support experimental translation of predicted biological effects.

Beyond pharmacokinetic considerations, certain methodological limitations should be acknowledged, most notably the focused analysis of four representative constituents. While PC contains a diverse array of minor and trace compounds, we prioritized these Core-4 markers because they represent well-established chemical fingerprints and major pharmacologically active stilbenes and anthraquinones. This focused strategy enabled a clearer characterization of dominant mechanistic pathways while minimizing potential confounding effects from poorly characterized constituents with uncertain bioavailability. In addition, network centrality reflects topological prominence within predicted compound–target architectures and should not be interpreted as direct evidence of causal importance. To enhance biological plausibility, disease-derived transcriptomic datasets were therefore integrated to assess whether predicted targets aligned with transcriptional signatures observed in insulin-resistant adipose tissue and atherosclerotic lesions.

Although shared targets across chemically distinct constituents suggest potential cooperative pathway modulation, no experimental multivariate synergy assay was performed. Accordingly, the proposed multi-component interactions should be interpreted as hypothesis-generating. Experimental validation will ultimately be required to determine whether cooperative effects predicted in silico translate into measurable biological interactions.

**Figure 10:**
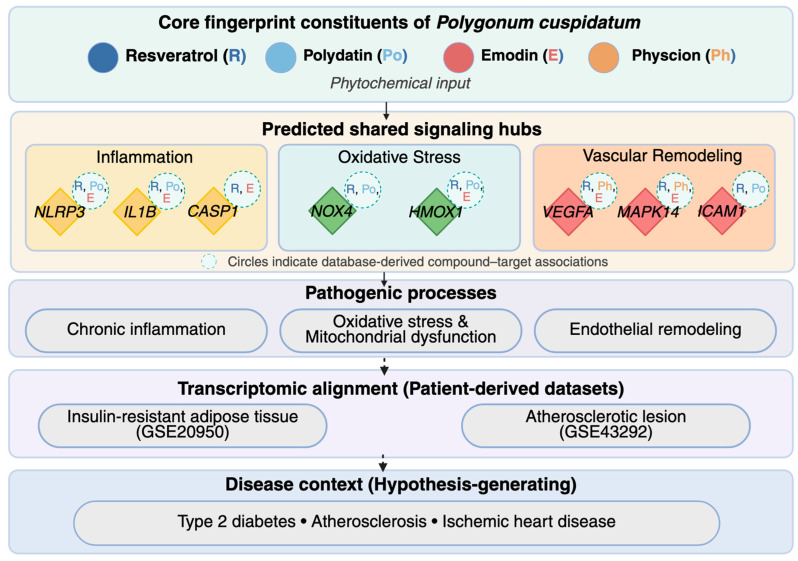
Systems-level framework linking core *Polygonum cuspidatum* constituents to metabolic and vascular disease pathways. Core fingerprint constituents of *Polygonum cuspidatum* (resveratrol, polydatin, emodin, and physcion) were mapped to predicted compound–target interactions derived from network pharmacology analysis. Convergent regulatory hubs associated with inflammation, oxidative stress, and vascular remodeling were identified and organized into interconnected pathogenic processes. Alignment with patient-derived transcriptomic datasets from insulin-resistant adipose tissue (GSE20950) and atherosclerotic lesions (GSE43292) supports the disease relevance of the predicted framework. Circles indicate database-derived compound–target associations.

Unlike earlier studies focused on isolated constituents, this work integrates phytochemical fingerprint selection, network pharmacology, and patient-derived transcriptomic evidence within a unified analytical framework. Rather than proposing single-target mechanisms, our findings support coordinated modulation across interconnected pathogenic pathways linking insulin resistance, chronic inflammation, oxidative stress, and vascular remodeling (Figure 10). As an exploratory in silico investigation, this system-level framework provides a rational basis for future targeted experimental studies investigating the biological relevance of PC in metabolic and vascular disease models.

## Figures and Tables

**Table 1:** ADME-related physicochemical properties of four representative marker compounds of *Polygonum cuspidatum*.

Compound	Chemical Class	MW (g/mol)	OB (%)	Caco-2	DL
Resveratrol	Stilbene	228.26	19.07	0.8	0.11
Polydatin	Stilbene glycoside	390.42	21.44	−0.9	0.5
Emodin	Anthraquinone	270.25	24.4	0.22	0.24
Physcion	Anthraquinone	284.28	22.29	0.52	0.27

These four compounds are widely recognized as the chemical fingerprint constituents of *Polygonum cuspidatum*. Abbreviations: MW, molecular weight; OB, oral bioavailability; Caco-2, Caco-2 cell permeability (an indicator of intestinal absorption potential); DL, drug-likeness.

**Table 2:** Quantitative chemical composition of major fingerprint compounds in different parts of *Polygonum cuspidatum*.

Plant Part	Polydatin	Resveratrol	Emodin	Physcion	References
Root	9.27–34.55	0.97–15.42	3.59–17.62	1.71–15.72	[32,33,34]
Stem	0.08–0.66	ND–0.15	ND–0.04	ND–0.08	[32]
Leaf	0.13–1.18	ND–0.13	ND–0.11	ND–0.39	[32]

ND: not detected (mg/g dry weight).

**Table 3:** Topological parameters of target genes in the compound–target network of *Polygonum cuspidatum*.

Gene Name	Betweenness Centrality	Degree Centrality
*NOS2*	0.05	4
*BAX*, *MAPK14*, *MAPT*, *PTGS2*	0.03	3
*NLRP3*, *NFKB1*, *HMOX1*, *GABPA*, *IL1B*, *VEGFA*, *TNF*, *PARP1*, *CTNNB1*, *SOD1*	0.01	3
*ACHE*	0.02	2
*VDR*, *GSK3B*, *PPARG*	0.006	2
*ICAM1*, *PPARGC1A*, *INS*, *NOX4*, *IL10*, *RUNX2*, *CDH1*, *STAT3*, *SIRT1*, *GPX4*, *VCAM1*, *GTF2H1*, *MUC5AC*, *SIRT3*, *METTL3*, *GPM6A*, *HDAC9*, *APCS*, *MIR139*, *AKT1*, *MUC2*, *MMP3*, *MARVELD1*, *TGFB1*, *CASP1*, *BCL2*, *IFNG*, *TP53*, *MAPK8*, *TJP1*, *MAPK1*, *SMAD3*, *SLC2A1*, *IFNB1*, *MTOR*, *TLR4*, *MAPK3*, *APP*, *CCL2*, *PPARA*, *KDR*, *IL6*	0.004	2
*MYC*	0.003	2

**Table 4:** DEG and Core-4 target (degree ≥ 2) intersections from GSE20950 and GSE43292 and their associated KEGG pathways related to metabolic and vascular regulation.

Dataset	Gene	Core-4 Compounds	log_2_FC	−log_10_ (*p*)	KEGG Pathways (Significant)
GSE20950	*MAPK14*	Emodin, Physcion, Resveratrol	−0.883	5.157	AGE–RAGE; lipid & atherosclerosis; TNF; IL-17; fluid shear stress & atherosclerosis; Toll-like receptor; NOD-like receptor; FoxO; MAPK signaling
	*MAPT*		−0.653	2.792	MAPK signaling
	*VEGFA*	Emodin, Polydatin, Resveratrol	−0.617	4.601	AGE–RAGE; fluid shear stress & atherosclerosis; HIF-1 signaling
	*PPARA*	Emodin, Resveratrol	−0.939	8.325	Insulin resistance; adipocytokine signaling
	*TGFB1*		−0.776	5.447	AGE–RAGE; FoxO; MAPK signaling
	*IFNG*		0.591	4.42	Fluid shear stress & atherosclerosis; HIF-1; IL-17 signaling
	*MAPK1*		−0.737	4.223	AGE–RAGE; lipid & atherosclerosis; HIF-1; TNF; IL-17; Toll-like receptor; NOD-like receptor; FOxO; MAPK signaling
	*BCL2*		−0.584	3.726	AGE–RAGE; lipid & atherosclerosis; HIF-1; fluid shear stress & atherosclerosis; NOD-like receptor
	*NOX4*	polydatin, resveratrol	−0.591	3.878	AGE–RAGE signaling
GSE43292	*IL1B*	Emodin, Polydatin, Resveratrol	0.78	2.27	AGE–RAGE; lipid & atherosclerosis; TNF; IL-17; fluid shear stress; MAPK; Toll-like receptor; NOD-like receptor signaling
	*NLRP3*		0.712	4.44	Lipid & atherosclerosis; NOD-like receptor signaling
	*HMOX1*		1.42	4.332	HIF-1; fluid shear stress signaling
	*NOX4*	Polydatin, Resveratrol	−0.882	4.585	AGE–RAGE
	*ICAM1*		0.754	3.744	AGE–RAGE; lipid & atherosclerosis; TNF; fluid shear stress signaling
	*CASP1*	Emodin, Resveratrol	0.635	3.871	Lipid & atherosclerosis; NOD-like receptor signaling
	*PPARG*	Emodin, Physcion	0.686	3.104	Lipid & atherosclerosis; insulin resistance; adipocytokine signaling

## Data Availability

All data used in this study are publicly available from open-access databases. Compound information was retrieved from TCMSP (https://tcmsp-e.com, accessed on 21 January 2026) and HERB 2.0 (http://herb.ac.cn/v2, accessed on 21 January 2026). Functional enrichment analyses were conducted using g:Profiler (https://biit.cs.ut.ee/gprofiler/gost, accessed on 23 Janueary 2026), DAVID (https://davidbioinformatics.nih.gov/, accessed on 23 January 2026), and the Genetic Association Database (GAD). Transcriptomic datasets were obtained from the Gene Expression Omnibus (GEO) under accession numbers GSE20950 and GSE43292 (https://www.ncbi.nlm.nih.gov/geo/, accessed on 23 January 2026). All analysis scripts used in this study are publicly available via a GitHub repository and have been archived on Zenodo with a persistent DOI (DOI: 10.5281/zenodo.18377571).

## Supplementary Materials

The following supporting information can be downloaded at: https://www.mdpi.com/article/10.3390/biomedicines14030516/s1, Figure S1: Disease enrichment analysis based on DisGeNET; Table S1: Lipinski’s rule-of-five compliance of the core-4 compounds of *Polygonum cuspidatum*; Table S2: Genes shared between the predicted targets of the core-4 compounds and DEGs from GSE20950 (adipose tissue, insulin-resistant vs. insulin-sensitive); Table S3: Genes shared between the predicted targets of the core-4 compounds and DEGs from GSE43292 (atheroma plaque vs. intact arterial tissue); Table S4: Global KEGG pathway enrichment results for DEGs from GSE20950 and GSE43292.

## Abbreviations

The following abbreviations are used in this manuscript:

PC	*Polygonum cuspidatum*
OB	Oral bioavailability
DL	Drug-likeness
MW	Molecular weight
Caco-2	Caco-2 cell permeability
ADME	Absorption, distribution, metabolism, and excretion
TCMSP	Traditional Chinese Medicine Systems Pharmacology database
GO	Gene Ontology
KEGG	Kyoto Encyclopedia of Genes and Genomes
GAD	Genetic Association Database
GEO	Gene Expression Omnibus
DEG	Differentially expressed gene
IR	Insulin resistance
IS	Insulin sensitive
FDR	False discovery rate
BH FDR	Benjamini–Hochberg false discovery rate
