# Supplementary material for: Network Pharmacology and Transcriptome Analysis Reveal Potential Cardiometabolic Targets of Polygonum cuspidatum
Source: Biomedicines. 2026 Feb 26;14(3):516. doi: 10.3390/biomedicines14030516 (PMC13023815; doi:10.3390/biomedicines14030516)
Supplement: Supplementary file 1 [file biomedicines-14-00516-s001.zip › Supplementary files.pdf]

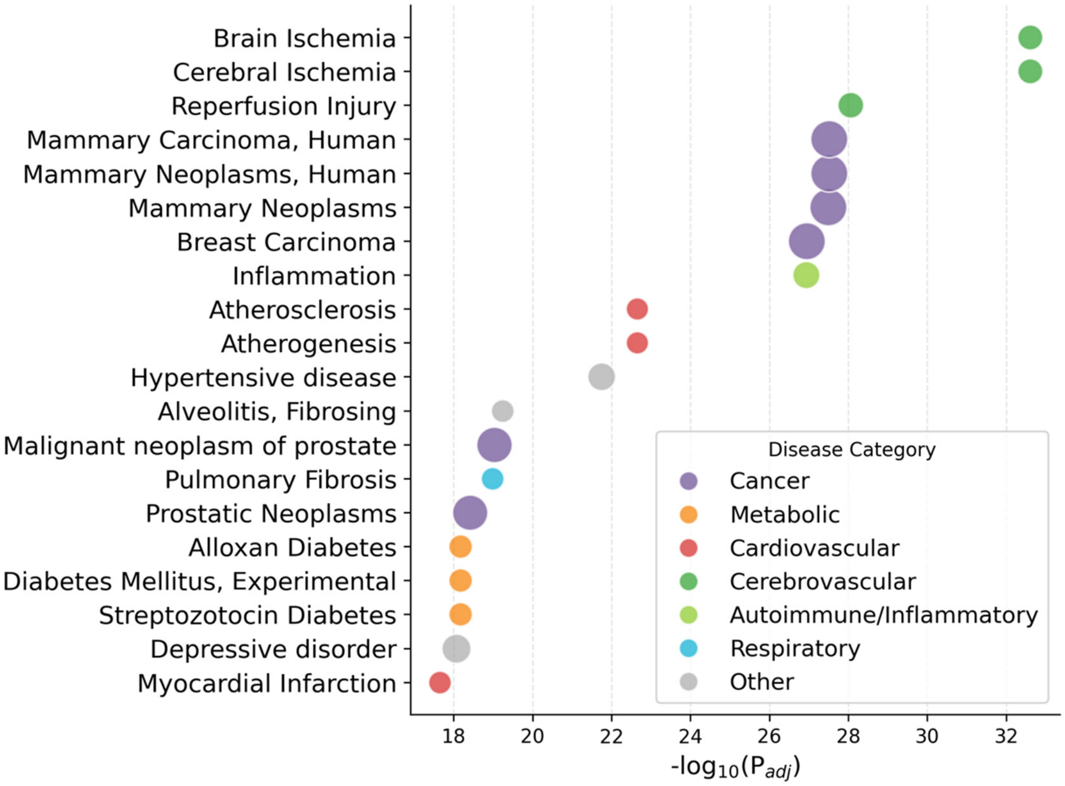

Supplementary Figure S1. Disease enrichment analysis based on DisGeNET.
The top 20 enriched disease terms associated with the predicted target genes of *Polygonum cuspidatum* were identified using the DisGeNET database. Diseases are ranked according to $-\log_{10}$(adjusted *P* values), and node size reflects the number of associated target genes.

Supplementary Table S1. Lipinski's rule-of-five compliance of the core-4 compounds of *Polygonum cuspidatum*.

Compound	MW < 500	logP < 5	HBD ≤ 5	HBA ≤ 10	Ro5 violations	Ro5 compliant
Resveratrol	✓	✓	✓	✓	0	Yes
Polydatin	✓	✓	✗	✓	1	No
Emodin	✓	✓	✓	✓	0	Yes
Physcion	✓	✓	✓	✓	0	Yes

Abbreviations: MW, molecular weight; logP, octanol–water partition coefficient; HBD, hydrogen bond donors; HBA, hydrogen bond acceptors; Ro5, Lipinski's rule-of-five.

Supplementary Table S2. Genes shared between the predicted targets of the core-4 compounds and DEGs from GSE20950 (adipose tissue, insulin-resistant vs. insulin-sensitive).

Gene Symbol	Core-4 Compounds	Degree	Log2(Fold Change)	-log10(adj P)
MAPK14	Emodin, Physcion, Resveratrol	3	-0.883	4.091
MAPT			-0.653	2.215
VEGFA	Emodin, Polydatin, Resveratrol	3	-0.617	3.652
PPARA	Emodin, Resveratrol	2	-0.939	8.325
APP			-0.686	6.452
TGFB1			-0.776	5.447

IFNG			0.591	4.42
MAPK1			-0.737	4.223
BCL2			-0.584	3.726
NOX4	Polydatin, Resveratrol	2	-0.591	3.079
CYP3A4	Resveratrol	1	-1.295	16.438
NRF1			-0.977	11.777
SPDEF			-0.649	7.996
MDM2			-0.622	5.951
CDKN2A			-0.689	5.402
NPPA			-0.614	5.312
PRKDC			-0.79	4.907
ATM			0.699	4.463
CNR1			-0.698	3.646
HIF3A			-0.905	3.178
HGF			0.775	3.067
CREB1			-0.696	2.917
STAT1			-0.814	2.295
CDH2	Polydatin	1	-0.61	3.099
ZEB1			-0.703	2.783
F7	Physcion	1	-0.85	9.206
EPHX2			-0.721	7.824
PGD			-0.616	6.328
NCOA2			-0.673	3.583
PTGS1			-0.942	2.072
XRCC1	Emodin	1	-0.972	11.359
FLT1			-0.583	6.714
DLG4			-0.671	4.102
SMAD2			-0.649	3.804
UCP1			-0.625	2.227

Supplementary Table S3. Genes shared between the predicted targets of the core-4 compounds and DEGs from GSE43292 (atheroma plaque vs. intact arterial tissue).

Gene Symbol	Core-4 Compounds	Degree	Log2(Fold Change)	-log10(adj P)
NLRP3	Emodin, Polydatin, Resveratrol	3	0.712	4.44
HMOX1			1.42	4.332
IL1B			0.78	2.27
NOX4	Polydatin, Resveratrol	2	-0.882	4.585
ICAM1			0.754	3.744
CASP1	Emodin, Resveratrol	2	0.635	3.871
PPARG	Emodin, Physcion	2	0.686	3.104
FABP4	Resveratrol	1	2.454	4.627
CD163			1.131	4.516
IL1RN			1.394	4.269
LEF1			0.618	4.201
SYK			0.856	4.188
GPX1			0.653	3.978
PECAM1			0.766	3.977
CD86			0.888	3.881

LRP6			-0.625	3.83
TREM2			0.646	3.805
TNFRSF1B			0.709	3.786
MMP9			1.818	3.746
MRC1			0.86	3.609
BDNF			-0.649	3.597
NR1H3			0.766	3.476
SLC2A3			0.784	3.396
LYZ			0.898	3.298
MKI67			0.629	3.086
IL18			0.805	3.063
CCL3			0.725	2.658
FPR1			0.756	2.554
ZEB1	Polydatin	1	-0.669	4.559
RYR2			-1.241	4.271
SELE			1.157	3.612
SPP1			0.733	2.387
DPP4	Physcion	1	1.611	4.442
PGD			0.941	4.409
PTGS1			0.589	4.124
THRB			-1.037	3.946
CA2			0.791	3.728
GLA			0.611	3.486
HPGD			0.76	2.486
ITGAM	Emodin	1	0.993	4.14
PRKG1			-0.839	3.887
CD36			1.802	3.796
FLT1			0.752	3.792
ALOX5			0.75	3.687
TNFRSF9			0.669	2.799

Supplementary Table S4. Global KEGG pathway enrichment results for DEGs from GSE20950 and GSE43292. (provided as a separate file due to size)
